# Benchmarking Alzheimer’s disease prediction: personalised risk assessment using polygenic risk scores across various methodologies and genome-wide studies

**DOI:** 10.1186/s13195-024-01664-9

**Published:** 2025-01-06

**Authors:** Eftychia Bellou, Woori Kim, Ganna Leonenko, Feifei Tao, Emily Simmonds, Ying Wu, Niklas Mattsson-Carlgren, Oskar Hansson, Michael W. Nagle, Valentina Escott-Price, the Alzheimer’s Disease Neuroimaging Initiative

**Affiliations:** 1 UK Dementia Research Institute at Cardiff, Cardiff University, Hadyn Ellis Building, Maindy Road, Cardiff, CF24 4HQ UK; 2 Eisai Inc, Cambridge, MA 02140 USA; 3 Clinical Memory Research Unit, Department of Clinical Sciences Malmö, Faculty of Medicine, Lund University, Lund, 221 00 Sweden; 4 Memory Clinic, Skåne University Hospital, Malmö, 214 28 Sweden; 5 Division of Psychological Medicine and Clinical Neurosciences, School of Medicine, Cardiff University, Hadyn Ellis Building, Maindy RD, Cardiff, CF24 4HQ UK

**Keywords:** Alzheimer’s disease, Polygenic risk score, Risk prediction

## Abstract

**Background:**

The success of selecting high risk or early-stage Alzheimer’s disease individuals for the delivery of clinical trials depends on the design and the appropriate recruitment of participants. Polygenic risk scores (PRS) show potential for identifying individuals at risk for Alzheimer’s disease (AD). Our study comprehensively examines AD PRS utility using various methods and models.

**Methods:**

We compared the PRS prediction accuracy in ADNI (*N* = 568) and BioFINDER (*N* = 766) cohorts using five disease risk modelling approaches, three PRS derivation methods, two AD genome-wide association study (GWAS) statistics and two sets of SNPs: the whole genome and microglia-selective regions only.

**Results:**

The best prediction accuracy was achieved when modelling genetic risk by using two predictors: *APOE* and remaining PRS (AUC = 0.72–0.76). Microglial PRS showed comparable accuracy to the whole genome (AUC = 0.71–0.74). The individuals’ risk scores differed substantially, with the largest discrepancies (up to 70%) attributable to the GWAS statistics used.

**Conclusions:**

Our work benchmarks the best PRS derivation and modelling strategies for AD genetic prediction.

**Supplementary Information:**

The online version contains supplementary material available at 10.1186/s13195-024-01664-9.

## Background

Alzheimer’s disease (AD) is the most common neurodegenerative disease that is caused by a combination of multiple genetic, lifestyle, and environmental factors. With an ageing population, the number of individuals with late-onset AD, and the economic burdens associated with their care, is rapidly growing [1]. Given recent regulatory approvals for therapies designed to treat earlier stages of AD [2], better strategies are needed to increase the efficiency of clinical trials [3]. For clinical trials to be successful, an appropriately sized and clinically characterized cohort is required [4], enrolment of which remains one of the most challenging aspects of the process. As AD pathology starts long before onset of symptoms, diagnosis of the disease at earlier stages is highly desired. For this reason, predictive biomarkers [5] are commonly used for reliably identifying patients with mild cognitive impairment who would be at higher risk of progressing to AD [6].

The use of an individual’s genetic information may provide complementary inclusion criteria to enhance clinical trial efficiencies [7]. Although *APOE*-e4 is the strongest common risk variant for late-onset of AD, its prediction accuracy is limited [8], and other variants discovered though genome-wide association studies (GWAS) have small individual effects [9, 10]. Polygenic risk scores (PRS) [11] which aggregate the small effects from individual variants across the genome into a single score have been shown to potentially be better stratification tools to identify those individuals at higher risk of AD [12]. However, a wide variability in the prediction accuracy of PRSs has been observed depending on cohort types (population-based or case/control study), definition of AD (clinical, pathological, self-reported, proxy); ancestry group (European, non-European or admixed), age of cases and controls, and other factors. Therefore, clinical interpretation of PRS is still a very challenging task.

In this study we aimed to comprehensively examine AD risk modelling strategies based on genetic information and provide clear recommendations and interpretation that can be used for selecting high risk or early-stage AD individuals for the delivery of clinical trials. We have chosen to use three PRS calculation methods which capture the main principles of commonly used PRS calculation approaches: clumping and thresholding PRS(C + T) [13, 14], PRS-CS [15] (used with two different LD reference panels) and quickPRS (a faster version of MegaPRS) [16]. We assessed these methods across two independent cohort studies: the Alzheimer’s Disease Neuroimaging Initiative (ADNI) and the Biomarkers for identifying Neurodegenerative Disorders Early and Reliably (BioFINDER). We compared the prediction performance of PRS using SNP effect sizes from two of the latest AD GWAS: clinically assessed [9] (AD-PRS), and a meta-analysis European proxy-AD GWAS which may better reflect AD-related dementia [10] (ADRD-PRS). Moreover, we tested different prediction modelling strategies, using the whole-genome PRS, as well as including and excluding *APOE* region. As microglia play a very important part in regulation of neuroinflammation in AD [17, 18] and can explain a substantial proportion of AD heritability [19], the same models and approaches were used to explored prediction utility of PRS based on genomic regions categorized as “microglia-selective”.

Finally, we investigated consistency of individuals’ scores derived with the above-mentioned strategies assessing the overlap of individuals who are at “high” or “low” AD risk.

## Methods

### Cohorts

#### Discovery cohorts

Two GWAS summary statistics data were used to generate polygenic scores: (a) a GWAS [9] that was an extension to the International Genomics of Alzheimer’s Project (IGAP) [20] with 21,982 clinically diagnosed AD cases and 41,944 controls (AD) and (b) a meta-analyzed GWAS [10] of the European Alzheimer & Dementia Biobank (EADB) consortium dataset with the UK Biobank (UKBB) data including 39,106 clinically diagnosed AD cases, 46,828 proxy-AD cases and 401,577 population-based controls (ADRD).

#### Target cohorts

##### ADNI123

ADNI is a longitudinal study that was developed for the early detection of AD with the use of clinical, genetic, and imaging data [21]. The data were collected from participants between ages 55 and 90 years. Initially, participants were followed for 2 to 3 years with repeated imaging scans and psychometric measurements (ADNI1). The study was extended with the addition of new participants (ADNI-GO, ADNI2 and ADNI3). All participants provided written consent. More information can be found at http://adni.loni.usc.edu/.

The ADNI samples were genotyped in three stages: ADNI1 (757), ADNI2-GO (432) and ADNI3 (327) (we call the combined dataset “ADNI123” thereafter) and standard quality control (QC) analysis was performed [22] using PLINK (https://www.cog-genomics.org/plink2) [23]. Initial QC was performed on each cohort separately removing individuals who did not cluster near the 1000 Genomes Project (1000G) [24] European population and removing SNPs with MAF < 0.01, Hardy-Weinberg Equilibrium (HWE) (*p* $\:\le\:$ 10^−6^) and with missing data rate>5%. Each cohort was imputed using TOPMED panel (https://imputation.biodatacatalyst.nhlbi.nih.gov/) and further combined and re-QCed. SNPs were removed with poor accuracy of imputation (INFO<0.7), MAF<0.05, missingness>5%, HWE$\:\le\:$ 10^−6^ and individuals checked for relatedness (Pi^hat > 0.2), leaving 1,361 individuals and 8,718,573 SNPs for our further analysis. Eight principal components (PCs) were calculated with PLINK and used for PRS analysis. Finally, 441 participants were removed from the analysis due to the overlap with the IGAP consortium data [9], which was part of the summary statistics. All variants were aligned to GRCh38/hg38.

To retain maximum sample size and the most accurate diagnosis, we used age and clinical diagnosis at the last assessment, leaving 223 AD cases and 345 healthy controls with available phenotypic and genetic information for the PRS analysis (see Table 1).

**Table 1:** Characteristics and demographics in ADNI123 and BioFINDER studies

Characteristics	ADNI123			BioFINDER		
	AD (*N* = 223)	Controls (*N* = 345)	*P*-value	AD (*N* = 170)	Controls (*N* = 596)	*P*-value
Age: Mean (SD)	77.6 (8.9)	75.1 (7.1)	< 0.001	74.8 (8.00)	72.1 (5.7)	< 0.001
Sex: Female N (%)	88 (39.6)	197 (57.1)	< 0.001	106 (62.4)	376 (63.1)	0.932
*APOE*-ε2 carrier: N (%)	10 (4.5)	38 (11.0)	0.006	9 (5.3)	92 (15.4)	0.001
APOE-ε4 carrier: N (%)	139 (62.3)	111 (32.1)	< 0.001	112 (65.9)	191 (32.0)	< 0.001
APOE-ε3ε3 carrier: N (%)	77 (34.5)	200 (58.0)	< 0.001	52 (31.1)	326 (55.0)	< 0.001
Site:						
Lund N (%)	N/A	N/A	N/A	0 (0)	46 (7.7)	N/A
Malmo N (%)	N/A	N/A	N/A	170 (100)	550 (92.3)	< 0.001

*AD* Alzheimer’s Disease, *N* sample size, *SD* Standard Deviation

##### BioFINDER

The BioFINDER study is an ongoing multi-centre longitudinal cohort study from the Swedish population with more than 1,600 patients with mild cognitive symptoms, dementia, and parkinsonian symptoms as well as cognitively healthy elderly [25, 26]. The subjects underwent detailed clinical assessments, neuropsychological examinations, and amyloid and tau PET imaging. The BioFINDER subjects were genotyped using Illumina platform GSA-MDA v2 array. We used KING 2.2.5 software (https://kingrelatedness.com) to perform QC of the raw genotypes containing 1,531 subjects and 694,945 variants [27]. First, based on subject or genotype missingness greater than 5% and sex mismatch, we excluded 126,334 variants and 7 subjects. Subsequently, we checked the relatedness and excluded 19 relatives up to the first-degree based on the kinship coefficient threshold greater than 0.125. We assessed the genetic ancestry by calculating PCs with 1000G [24] and removed 2 individuals of non-European ancestry. Furthermore, 992 variants violating HWE (p $\:\le\:$10^−6^) were removed. After QC, we retained 1,503 subjects and 567,519 variants. The genotype data was further imputed using TOPMED reference panel (*GRCh38/hg38)* and the variants filtered based on INFO imputation accuracy score ≥ 0.3, HWE (*p* ≤ 10^−6^) and MAF > 0.005. Finally, we obtained 1,503 subjects and 9,991,979 variants. In this study, we included 170 AD cases and 596 healthy controls recruited at baseline, totaling 766 subjects (see Table 1). This sample was not part of the discovery summary statistics cohort.

### PRS methods

#### C + T

Both cohorts were LD-clumping with PLINK [23] by retaining the variant with the smallest *p*-value from each LD block and excluding variants with r^2^ > 0.1 in 1000-kb window. For both datasets we tested a range of *p*-value thresholds (pT = 5 × 10^−8^, 10^−6^, 10^−5^, 10^−3^, 10^−2^, 0.05, 0.1, 0.5) applied to AD and ADRD summary statistics and determined the optimal *p*-value threshold corresponding to the highest Area Under the ROC Curve (AUC) achieved.

#### PRS-CS

PRS-CS utilizes a high-dimensional Bayesian regression framework by placing a continuous shrinkage (CS) prior on effect sizes, which is robust to varying genetic architectures, and enables multivariate modelling of local LD patterns [15]. We ran PRS-CS using both 1000G and UKBB reference European ancestry panels. We ran the analysis with the automated optimisation model parameter (phi) representing global shrinkage. The other parameters have been set to the default option.

#### quickPRS

QuickPRS (https://dougspeed.com/quick-prs/) is an approximate version of MegaPRS [16] that implements LDAK model [28] with various summary statistics tools including BayesR [29] and assumes non-equal contribution of SNPs to the heritability of a trait, as compared to the previous models like GCTA and LDSC [30]. We used quickPRS with default settings and the pre-computed SNP-SNP correlation from a sample of 2,000 white British individuals, and pre-computed files corresponding to 1.0–1.2 M non-ambiguous HapMap3 SNPs. First, per-SNP heritability was estimated using the command --sum-hers along with commands --check-sums NO and --cutoff 0.01. Then, the prediction models were constructed using the command --mega-prs along with commands --model bayesr, --cv-proportion 0.1, and --window-cm 1 as suggested by the creators of the software.

### SNP selection

PRSs were calculated for all available SNPs (PRS_FULL_) and excluding SNPs physically located in the *APOE* region (CHR19: 43.9–46.0Mb (*GRCh38/hg38*), PRS_noAPOEregion_). If the PRS method used pre-calculated LD maps based on a particular reference panel, the list of SNPs was restricted to these variants. The same PRS approaches with the same parameters were used when we calculated PRS for microglial-selective regions as defined by GWAS signatures and epigenetic/gene regulatory data [17] and re-defined to include SNPs in established regulatory regions of genes determined from microglial ATAC-Seq data [18]. SNPs were extracted from these regions in each dataset separately to ensure the best coverage in terms of SNP availability and quality. The list of regions and SNPs can be found in [19], where it was also shown that these regions can explain a substantial proportion of AD heritability. It should be noted that the list of microglial-selective regions did not contain the *APOE* 4 allele risk SNPs but did contain SNPs from the *APOE* region.

### Statistical analyses

#### Descriptive analysis

Descriptive statistics were used to summarize the samples’ characteristics. Results are presented as mean and standard deviation (SD) for continuous variables, and number of participants (%) for categorical variables. The significance of between group differences was examined using the Mann-Whitney U-test or t-test for continuous variables and the chi-squared test for categorical variables. Results were reported as significant if *p*-value ≤ 0.05.

#### Evaluation of prediction performance/modelling approaches

All genetic scores (whole-genome and microglia) and *APOE* ε2 + ε4 counts generated by various PRS methods and models were adjusted for age, sex, and PCs and then standardized. The association of PRS with AD case/control status was examined using logistic regression in the following five models:

$$\begin{array}{c}\mathrm{Model}\;1\;(\mathrm M1):\;\mathrm{AD}\;\sim\;APOE(\mathrm\varepsilon2+\mathrm\varepsilon4)\\\mathrm{Model}\;2\;(\mathrm M2):\;\mathrm{AD}\;\sim\;{\mathrm{PRS}}_{\mathrm{APOEregion}}\\\mathrm{Model}\;3\;(\mathrm M3):\;\mathrm{AD}\;\sim\;{\mathrm{PRS}}_{\mathrm{FULL}}\\\mathrm{Model}\;4\;(\mathrm M4):\;\mathrm{AD}\;\sim\;{\mathrm{PRS}}_{\mathrm{noAPOEregion}}\\\mathrm{Model}\;5\;(\mathrm M5):\;\mathrm{AD}\;\sim\;{\mathrm{PRS}}_{\mathrm{noAPOEregion}}\;+\mathit\;APOE(\mathrm\varepsilon2+\mathrm\varepsilon4)\end{array}$$

where *APOE*(ε2 + ε4) is the numbers of *APOE* ε2 and ε4 alleles that were directly included in the prediction models. We report the accuracy of the models in terms of Area under the ROC Curve (AUC) as well as Beta coefficients (effect sizes) and their *p*-values. We tested M3 model in sub-samples of *APOE*(ε3ε3) carriers. We tested the accuracy of AD risk prediction by the PRS for microglial-selective regions using models M1-M5. We accounted for multiple testing using Bonferroni corrected significance threshold *p* ≤ 0.05/80 = 0.000625 within each datasets analysis, accounting for 80 tests: 5 (models) x 2 (summary statistics) x 4 approaches + 1 model (for e33 carriers) x 2 (summary statistics) x 4 approaches + 4 models (for microglia PRS) x 2 (summary statistics) x 4 approaches.

#### Comparison of risk scores among the methods and in individuals at extremes of PRS distributions

First, we performed a pairwise comparison of PRS scores from (M3) model, generated using AD and ADRD summary statistics, and computed Pearson correlations between them for both the whole-genome and microglia-selective regions.

Next, we investigated the consistency of individuals’ PRS scores in the first (left tail or low PRS) and the fifth (right tail or high PRS) quintiles (5 consequent groups with 20% of the samples in each) and report the percent of overlapping individuals that fall in the same quintile. We quantify the consistency by the percentage of overlapping individuals in 1st and 5th quintiles that were calculated with (a) the above PRS derivation approaches, (b) models M3 and M5 (utilizing the whole genome PRS and modelling the whole genome PRS by two variables, respectively), and (c) two AD GWAS summary statistics (AD and ADRD).

## Results

### Demographic and clinical characteristics for ADNI123 and BioFINDER

ADNI123 had 223 AD cases and 345 healthy controls at the last point of assessment with available genetic information. Their demographic and clinical characteristics are shown in Table 1. The sex distribution in ADNI123 was significantly different with more females in controls (39.6% vs. 57.1% in cases and controls respectively, *p* < 0.001). Statistically significant differences were also observed in age (77.6 years vs. 75.1 years in cases and controls respectively, *p* < 0.001). As expected, the proportion of ε2 carriers in AD cases was significantly lower compared to controls (4.5% vs. 11%, *p* = 0.006); whereas the proportion of ε4 carriers was significantly higher in cases (62.3% vs. 32.1%, *p* < 0.001) as compared to controls.

The BioFINDER sample consisted of 170 AD cases and 596 healthy controls as seen in Table 1. The AD cases were significantly older than the controls (74.8 years vs. 72.1 years, *p* < 0.001) with the sex distribution being similar for both groups. The AD cases in BioFINDER were recruited from one site, Malmo, while the controls were recruited from both the Malmo and Lund sites. The AD cases showed a significantly lower proportion of *APOE* ε2 carriers (5.3% vs. 15.4%, *p* < 0.001) and higher proportion of.

### Whole genome PRS

Using the PRS(C + T) approach we evaluated performance across a range of *p*-value thresholds in the ADNI123 and BioFINDER datasets. The analysis was conducted using AD and ADRD summary statistics in models M3, M4 and M5, see Supplementary Tables 1, 2 (A). For our further analysis, we selected the optimal thresholds based on AUC performance in M5. For ADNI123, the optimal pT was 0.1, achieving AUC = 0.72 for AD and 0.7 for ADRD summary statistics. For BioFINDER, the optimal pT was 5 × 10^−8^, yielding AUC = 0.74 for both AD and ADRD.

**Fig. 1:**
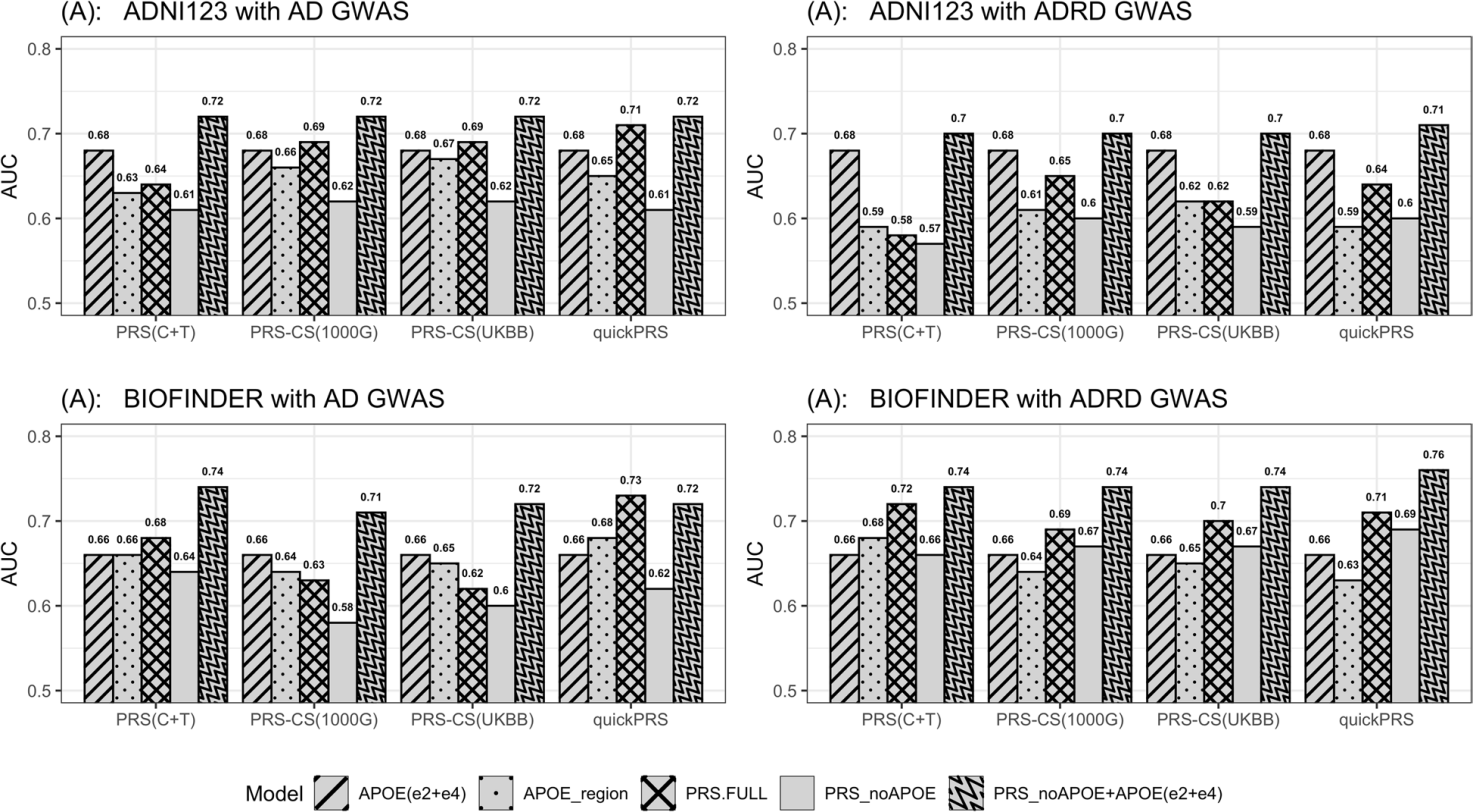
Prediction accuracies of the five models tested in ADNI123 and BioFINDER datasets with PRS calculation methods: PRS(C + T), PRS-CS(1000G), PRS-CS(UKBB) and quickPRS using genetic variants from AD [9] and ADRD [10] summary statistics

Figure 1 presents the prediction accuracies (AUCs) of each of the PRS approaches: PRS(C + T), PRS-CS (1000G), PRS-CS (UKBB) and quickPRS using genetic variants across the genome from the latest summary statistics AD [9] and ADRD [10] with M1-M5 models participants from ADNI123 (A, B) and BioFINDER (C, D), respectively. More details can be found in Supplemental Tables 3 and 4.

The best performing model is M5, which includes *APOE*(ε2 + ε4) as a separate variable in addition to PRS, excluding the *APOE* region (PRS_noAPOEregion_). This model has improved the prediction accuracy of AD risk over the model including only *APOE* alleles by maximum 10% (absolute increase). For example, model M5 using quickPRS method produced AUC of 0.71 and 0.76, while model M1 returned an AUC of 0.68 and 0.66 for ADNI123 and BioFINDER, respectively when using ADRD summary statistics (Fig. 1, plots (B) and (D)). In most cases the inclusion of *APOE* counts of alleles ε2 and ε4 returned consistently higher accuracy than the inclusion of *APOE* region (as a whole) for both ADNI123 and BioFINDER (see M1 versus M2 in Fig. 1). This due to the fact that these are two alleles defining the *APOE* status and the additional variants in the region are likely contributing to the noise. The lowest prediction accuracy was observed consistently in M4 for PRS without *APOE* region, with a max AUC = 0.62 and 0.69 in ADNI123 and BioFINDER, respectively (see Supplementary Tables 3 and 4).

When comparing results between ADRD vs. AD summary statistics, in ADNI123 slightly higher accuracies were achieved using AD summary statistics with the maximum AUC = 0.72 with M5 (see Fig. 1 (A) and (B)). In contrast, in BioFINDER the best performing GWAS was ADRD (max AUC = 0.76), see Fig. 1 (C) and (D).

Next, we set to investigate prediction accuracies using model M3 in individuals with *APOE*(ε3ε3) genotype. In this case 77 AD cases and 200 healthy controls were present in ADNI123 and 52 cases and 326 controls in BioFINDER. Overall, PRS prediction accuracies were reduced and achieved maximum of AUC = 0.64 (AD association *p* ≤ 2.3 × 10^−4^) in ADNI123 using AD summary statistics and PRS-CS (UKBB) approach. In BioFINDER the maximum AUC was 0.65 (AD association *p* = 6.1 × 10^−4^), using ADRD GWAS and PRS(C + T) approach (Supplementary Tables 3 and 4).

### Microglia-specific PRS

For this analysis, we have chosen specific microglia regions that have been derived and described in [17, 18]. Performance of PRS(C + T) approach was assessed across the range of *p*-values described above (see Supplementary Table 1(B) and 2 (B)). As above, for ADNI123, the optimal pT was 0.1 with AUC = 0.71 for AD and AUC = 0.69 for ADRD and for BioFINDER, the optimal pT was 1 × 10^−6^ with AUC = 0.72 for AD and AUC = 0.73 for ADRD.

With the utilization of SNPs that represent microglia-selective regions, the accuracies ranged from AUC = 0.60 to 0.67 in ADNI123 and BioFINDER using either AD or ADRD summary data (Supplementary Tables 5–6, Supplementary Fig. 1). The prediction accuracy without including *APOE* region was further reduced (compare M3 and M4). When the *APOE* gene was included (M5), the prediction accuracies recovered to almost full genome level PRS (0.67 to 0.74 depending on the PRS calculation approach).

### Benchmarking of the PRS results

**Fig. 2:**
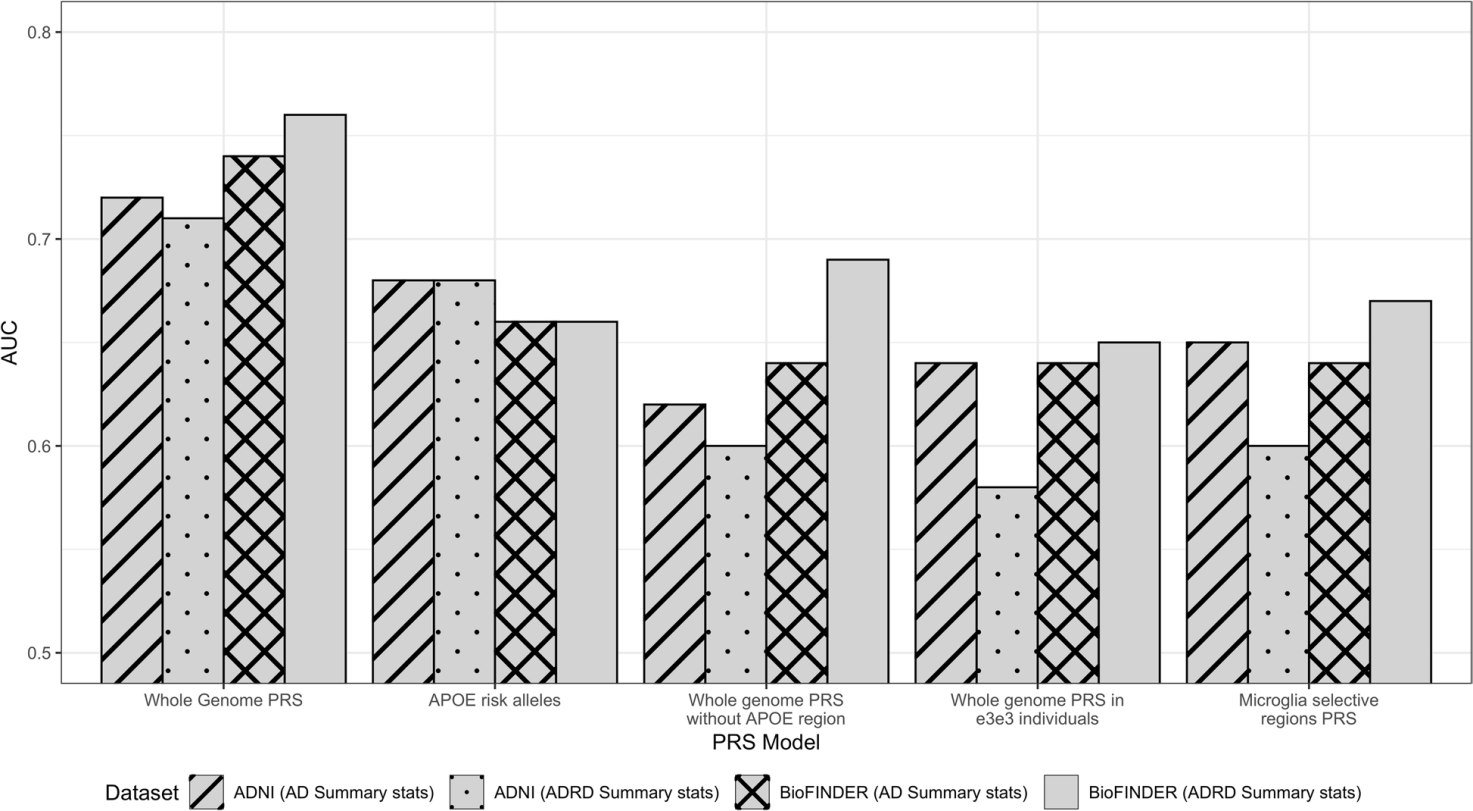
Comparison of the best prediction accuracy (max AUC across PRS calculation approaches) between datasets (ADNI123 and BioFINDER) for (1) PRS calculated for the Whole genome, (2) APOE risk alleles, (3) whole genome PRS without APOE region, (4) whole genome PRS in e3e3 individuals, and (5) PRS calculated for microglia-selective regions

We visualized a summary of our results in bar plot (Fig. 2) for both datasets ADNI123 and BioFINDER. X-axis represents the five main approaches to genetic prediction of AD risk: the whole genome PRS, *APOE*(ε2 + ε4), whole genome PRS without *APOE* region, whole genome PRS for *APOE*(ε3ε3) individuals, and PRS based upon microglia-selective regions. Y-axis shows the maxim AUC achieved across the PRS calculation approaches.

We showed that the best prediction accuracy (AUC = 0.71–0.76) was achieved with PRS calculated on the whole genome with model M5. Prediction accuracies of *APOE*(ε2 + ε4) alone showed similar results in both cohorts (AUC = 0.66–0.68), while in model M4 that represents whole genome without *APOE* region produced AUC in a range of 0.60–0.69 depending on the PRS methodology and GWAS used. PRS prediction accuracies calculated in individuals with *APOE*(ε3ε3) haplotype were reduced and achieved maximum of AUC = 0.65. Finally, PRS that was calculated on SNPs mapping to microglia-selective regions for M3 model showed accuracies in range of AUC = 0.60–0.67.

### Comparison of individual’s genetic scores

Supplementary Figs. 2 and 3 represent pairwise comparison of individual’s scores calculated as PRS_FULL_ between PRS calculation approaches for ADNI123 and BioFINDER cohorts respectively. In both datasets the highest correlation of individuals’ scores (cor > 0.90) was achieved between PRS-CS (1000G) and PRS-CS (UKBB) when the same summary statistics is used. The largest discrepancies occur when PRS were generated with different summary statists. Specifically, the lowest correlation (cor = 0.22) was observed in ADNI123 with PRS(C + T) approach. In BioFINDER, the lowest correlation (cor = 0.30) was noted with PRS-CS (UKBB) and QuickPRS approaches. A similar correlation pattern was observed when the scores were generated for microglial-selective regions, see Supplementary Figs. 4 and 5.

Finally, we quantified the consistency between an individual’s PRS by the proportion of overlap between people with high/low AD risk calculated with PRS(C + T), PRS-CS (1000G), PRS-CS (UKBB) and quickPRS approaches, Supplementary Tables 7 and 8. For that, we split individuals based upon PRS distribution into quintiles which resulted in 114 and 153 people in each quintile, in ADNI123 and BioFINDER, respectively. We report the percentage of overlapping individuals in the 1st (low risk) and 5th (high risk) quintiles.

First, we compared the agreement between *PRS calculation approaches* (fixing the models and summary statistics). In ADNI123, the percentage of overlapping individuals varied between 39 and 86%, with the highest consistency between PRS calculated with PRS-CS using two reference panels, and the lowest between PRS(C + T) and quickPRS, Supplementary Table 7.1. Model 5, where the *APOE* SNPs were included in addition to PRS_noAPOEregion_, has shown a systematically better consistency between individual’s scores (between 61 and 91%, Supplementary Table 7.2). A similar pattern of results was observed in BioFINDER, where the agreement between individual’s PRS scores ranged between 33 and 84% and 62–90% for M3 and M5, respectively (Supplementary Table 8.1 and 8.2).

We then compared the consistency between the individuals’ PRS scores, calculated with *the two (AD and ADRD) summary statistics*, now fixing the PRS calculation approach and the prediction model, Supplementary Table 7.3, 8.3. The percentage of overlapping individuals decreased ever further, ranging between 26 − 65%, whereas overall the prediction accuracy did not change (See Figs. 1 and 2).

Finally, we compared the agreement between *the risk modelling approaches*, i.e. the consistency between the PRS_FULL_ and a composite score using PRS_noAPOEregion_+ *APOE*(ε2 + ε4), Supplementary Table 7.4, 7.5, 8.4 and 8.5. The percentage of overlapping individuals varied between 37 and 78% in ADNI123 and between 38 and 72% in BioFINDER. The highest overlap has been achieved with the same PRS derivation approaches (diagonal values).

## Discussion

Remarkable progress has been made in understanding the genetics of AD, however more efforts are required to bring genetic testing into clinical medicine for public health benefits. PRS analysis, as one instrument of genetic testing, can play an important role in prevention strategies and identification of individuals at high risk of AD while also optimizing the design of clinical trials and/or help in long-term care planning. However, there is still a lack of agreement in practical implementation of polygenic scoring due to imprecision in genetics findings (i.e. identifying proxy SNPs which are in LD with the causal SNPs) and PRS analytical methodology uncertainties. The latter includes the choice of methodology, summary statistics from which to derive variant weights, and parameters to select related to the chosen PRS calculation approach. Moreover, PRS distributions are sample specific, i.e. the scores are not generally comparable between studies due to sample configuration (clinical vs. pathologically confirmed diagnoses, age, etc.), genotyping chips, imputation reference panels, and QC steps employed to generate the genetic data. Other PRS limitations include the predictive capacity that heavily depends on trait heritability (proportion of risk explained by SNPs) and population ancestry, where genetic data differs in LD structure, disease association SNP effect sizes, and allele frequencies [31, 32]. Some attempts have been made to integrate GWAS data from individuals of diverse ancestries alongside those data of the target population, showing a modest improvement in PRS risk prediction accuracy in diverse cohorts as compared with methods only utilizing GWAS data from a single ancestry source [32, 33]. Finally, there is a lack of independent AD genetic studies from which to test methods as most disease-specific cohorts are included in the latest/largest GWAS studies [34–36]. Because independence between subjects in GWAS studies and a target cohort is required for PRS calculation, even a small sample overlap may significantly inflate results [37].

Several publications [38] offer recommendations for best practice in PRS calculation, but this translates into very limited real-world clinical applications. In this study we set a benchmark for polygenic scoring with application to AD in European populations in terms of PRS methodologies, risk prediction modelling, and GWAS summary statistics which can help design future patient stratification and treatment strategies in clinical trials. For this, we used two independent studies (ADNI123 and BioFINDER), which were not included in the published GWAS.

We observed that the prediction accuracies in general stay similar for different PRS calculation approaches with variation about 0.02–0.06 within the same model. This study also confirms previous findings [12, 39] that the best accuracy is achieved with the model that includes *APOE*(ε2 + ε4) as a separate variable in addition to PRS which excludes the *APOE* region. This appears counterintuitive as there are more and more papers reporting different and inconsistent additional risk variants in this region beyond *APOE*-e4 and *APOE*-e2, however due to high LD in this region, the PRS calculation approaches have not yet separated biologically relevant SNPs from the noise. In our study, maximum prediction accuracy was achieved by this model with AUC = 0.72 and 0.76, in ADNI123 and BioFINDER, respectively. The PRS prediction accuracies in individuals with *APOE*(ε3ε3) genotype were achieved in ADNI123 using AD summary statistics and PRS-CS (UKBB) approach (AUC = 0.64), and with ADRD GWAS and quickPRS (AUC = 0.65) in BioFINDER, which is not sufficient to prioritize high risk individuals for clinical trials or other type of research. PRS based on microglia-selective regions produced accuracies comparable to the whole genome PRS, when *APOE* causal SNPs were included in the regression model (AUCs of 0.7–0.74 as compared 0.72–0.76 for whole genome). The interplay between microglia, *APOE* and ageing is still unclear, but probably have synergistic effects that contributes to the overall AD risk [40]. We find this observation important since microglia specific regions contain only 3% of variants of the whole genome, and therefore inclusion of a substantially smaller number of SNPs is likely to increase the consistency of PRS at the individual level.

When designing a study based on polygenic risk score, two key factors require careful consideration (1) the selection of GWAS summary statistics and (2) the choice of the PRS calculation method.

The preference of GWAS summary statistics often defaults to the latest and largest available datasets. However, the inclusion of UK Biobank (UKBB) participants in many recent GWAS, especially for predicting neurodegenerative disorders, has introduced challenges due to pervasive biases in the UKBB [41]. In our study, we observed small differences in predictive accuracy between AD and ADRD summary statistics. AD GWAS yielded slightly higher accuracies in the ADNI123 dataset, whereas ADRD GWAS produced better results in BioFINDER. These differences may reflect population-specific characteristics and differences in case-control ascertainment. For example, the BioFINDER dataset includes patients who later developed other forms of dementia, whereas ADNI123 is more focused on AD cases. Additionally, despite the much larger sample size of the ADRD GWAS (*N* ~ 500,000), the predictive accuracy for AD case/control status was comparable across all tested PRS derivation approaches.

PRS calculation method depends upon the purpose of the study, and usually involves parameters’ optimisation. For example, for the PRS(C + T) approach, we observed that the optimal *p*-value threshold varied between the datasets. In ADNI123, the optimal pT was 0.1, reflecting a polygenic genetic architecture of AD, while for BioFINDER the optimal threshold was 5 × 10⁻⁸, capturing only the genome-wide significant loci. In the latter, the prediction accuracy is nearly identical using either AD or ADRD summary statistics, as the genome-wide significant loci are largely shared between the two GWAS. This implies that genome-wide significant loci are likely transferable between European populations, such as the USA (ADNI123) and Sweden (BioFINDER). However, variants with smaller effects may provide better predictive power when the discovery and target datasets are from closely related populations (e.g., the majority of participants in both AD and ADRD GWAS are from the UK and USA).

Overall, our results show that prediction accuracy varied by a maximum of 0.06 in the absolute value of AUC, regardless of the methods or parameters used. This level of variation is not significant enough to be a critical consideration when designing a study.

Importantly, in classifying individuals based upon their PRS scores into high/low risk of AD (1st and 5th quintiles of the corresponding PRS distribution), we showed that the agreement between individuals’ scores varied substantially across all settings. Comparing the PRS derivation methods, on average the proportion of overlapping individuals was 50%, with the highest (over 90%) between PRS-CS with different reference panels. The model which includes the *APOE* risk alleles as a separate predictor has shown systematically higher consistency than other models. The largest discrepancies between individuals’ scores were produced when PRS were generated with two different summary statistics (AD and ADRD) with the average proportion of overlapping PRS extremes individuals of 39%. Some discrepancy between the individual’s PRS is expected even if the same SNPs are used. Indeed, SNP effect sizes (B-coefficients) estimated by a GWAS have certain variability measured by Standard Errors (SE). The variability for each SNP leads to further increased uncertainty in estimates of PRS value in a target individual when many SNPs are combined. The probability theory dictates that the individual PRS value, as a sum B-coefficients weighed by the number of risk alleles that the individual carries, is approximately normally distributed with this PRS value being the mean and the variance being accumulated in accordance with each SNP-related variance. In theory, increasingly large GWAS sample sizes are expected to provide more accurate B-coefficients estimates and reduce SEs. Ding et al. [42] provide an analytical estimator for the expectation of individual PRS variance as a function of SNP heritability and number of causal SNPs, however neither of these is certain in AD, with the larger GWAS showing lower SNP-based heritability [34]. It was expected that PRS calculated using a smaller number of variants, such as microglial PRS, would yield more consistent individual scores. However, this was not observed in our study (see Supplementary Figs. 4 and 5). This discrepancy suggests that the two GWAS datasets, which focus on different phenotypes (AD vs. Proxy AD), possess distinct underlying properties.

### Limitations

Several limitations need to be considered. First, both target cohorts have relatively small sample sizes and may exhibit different methods of case and control ascertainment. However, they are independent of each other and from the discovery summary statistics. The main conclusions are based on results from both studies, which can, in a way, be considered as replication. Next, our definition of the *APOE* region may not fully exclude all SNPs in linkage disequilibrium with *APOE* variants in every population. However, it is broader than those used in other AD studies of European descent, such as those by Lambert et al. [20] and Kunkle et al. [9]. Another limitation of our study is that it examines prediction accuracies based solely on genetic risk for AD, without considering factors such as age, gender, or other contributors to AD risk. A recent study by Lo et al. [43] highlights a potential difference in the genetic architecture of AD based on the age of clinical onset. Furthermore, disease-related mortality adds complexity to studies on the genetic background of neurodegenerative disorders. This makes PRS calculations that account for age particularly challenging due to the lack of age-stratified GWAS.

## Conclusions

We conclude that, while the prediction accuracy of PRS is generally consistent across different calculation approaches, the most significant variability in individuals’ PRS scores arises from the choice of GWAS summary statistics. Caution is advised when using these scores for screening or personal risk predictions. The selection of the PRS calculation method, SNPs/loci (whether cell-type specific or whole-genome), and discovery GWAS summary statistics should be carefully tailored to the specific context of the study.

## Supplementary Information

Supplementary Material 1.

## Data Availability

Kunkle et al. 2019 summary statistics for the Stage 1 can be obtained from The National Institute on Aging Genetics of Alzheimer’s Disease Data Storage Site (NIAGADS) - a NIA/NIH-sanctioned qualified-access data repository, under accession NG00075. Bellenguez et al, 2022 summary statistics has been deposited to European Bioinformatics Institute GWAS Catalog (https://www.ebi.ac.uk/gwas/) under accession no. GCST90027158. Initiative (ADNI) used in this study were available at the database (http://adni.loni.usc.edu/), upon registration and compliance with the data usage agreement. For BioFINDER data, anonymized data can be shared by request from a qualified academic investigator for the sole purpose of replicating procedures and results presented in the article and as long as data transfer is in agreement with EU legislation on the general data protection regulation and decisions by the Ethical Review Board of Sweden and Region Skåne, which should be regulated in a material transfer agreement.

## Abbreviations

AD: Alzheimer’s disease
ADNI123: combined dataset of ADNI1, ADNI2-GO and ADNI3
ADRD: Alzheimer’s disease related dementia
APOE4: Apolipoprotein E ε4
AUC: Area under the curve
EADB: European Alzheimer & Dementia Biobank consortium
GWAS: genome-wide association study
IGAP: International Genomics of Alzheimer’s Project
LD: linkage disequilibrium
PRS: Polygenic risk score
p: p-value
QC: Quality control
SNP: Single nucleotide polymorphism
UKBB: UK Biobank
1000G: 1000 Genomes Project

## References

[1] The State of Ageing in England is getting worse. Centre for Ageing Better. 2022. Available from: https://ageingbetter.org.uk/summary-state-ageing-2022.

[2] NIA statement on report of lecanemab reducing cognitive decline in Alzheimer’s clinical trial | National Institute on Aging. Available from: https://www.nia.nih.gov/news/nia-statement-report-lecanemab-reducing-cognitive-decline-alzheimers-clinical-trial. Cited 2023 Nov 8.

[3] Novitzke JM. The significance of clinical trials. J Vasc Interv Neurol. 2008;1:31 Available from: https://pubmed.ncbi.nlm.nih.gov/22518214/. Cited 2023 Nov 8. 22518214 PMC3317309

[4] Enrichment Strategies for Clinical Trials to Support Approval of Human. Drugs and Biological Products | FDA. Available from: https://www.fda.gov/regulatory-information/search-fda-guidance-documents/enrichment-strategies-clinical-trials-support-approval-human-drugs-and-biological-products. Cited 2023 Nov 8.

[5] Hansson O. Biomarkers for neurodegenerative diseases. Nat Med. 2021;27:954–63 Available from: https://pubmed.ncbi.nlm.nih.gov/34083813/. Cited 2024 Apr 19. 34083813 10.1038/s41591-021-01382-x

[6] Ballard C, Atri A, Boneva N, Cummings JL, Frölich L, Molinuevo JL, et al. Enrichment factors for clinical trials in mild-to-moderate Alzheimer’s disease. Alzheimer’s Dementia : Transl Res Clin Interv. 2019;5:164 Available from: https://pubmed.ncbi.nlm.nih.gov/31193334/. Cited 2023 Nov 8. 10.1016/j.trci.2019.04.001 PMC6527908 31193334

[7] Burke W, Psaty BM. Personalized medicine in the era of genomics. JAMA. 2007;298:1682–4 Available from: https://jamanetwork.com/journals/jama/fullarticle/209088. Cited 2023 Nov 9. 17925520 10.1001/jama.298.14.1682

[8] Escott-Price V, Sims R, Bannister C, Harold D, Vronskaya M, Majounie E, et al. Common polygenic variation enhances risk prediction for Alzheimer’s disease. Brain. 2015;138:3673–84. 26490334 10.1093/brain/awv268 PMC5006219

[9] Kunkle BW, Grenier-Boley B, Sims R, Bis JC, Damotte V, Naj AC, et al. Genetic meta-analysis of diagnosed Alzheimer’s disease identifies new risk loci and implicates Aβ, tau, immunity and lipid processing. Nat Genet. 2019;51:414–30. 30820047 10.1038/s41588-019-0358-2 PMC6463297

[10] Bellenguez C, Küçükali F, Jansen IE, Kleineidam L, Moreno-Grau S, Amin N, et al. New insights into the genetic etiology of Alzheimer’s disease and related dementias. Nat Genet. 2022;54:412–36. 35379992 10.1038/s41588-022-01024-z PMC9005347

[11] Purcell SM, Wray NR, Stone JL, Visscher PM, O’Donovan MC, Sullivan PF, et al. Common polygenic variation contributes to risk of schizophrenia and bipolar disorder. Nature. 2009;460:748–52. 10.1038/nature08185. Cited 2013 Sep 19. 19571811 10.1038/nature08185 PMC3912837

[12] Leonenko G, Baker E, Stevenson-Hoare J, Sierksma A, Fiers M, Williams J, et al. Identifying individuals with high risk of Alzheimer’s disease using polygenic risk scores. Nat Commun. 2021;12. Available from: https://pubmed.ncbi.nlm.nih.gov/34301930/. 10.1038/s41467-021-24082-z PMC8302739 34301930

[13] Wray NR, Goddard ME, Visscher PM. Prediction of individual genetic risk to disease from genome-wide association studies. Genome Res. 2007;17:1520–8 Available from: https://pubmed.ncbi.nlm.nih.gov/17785532/. Cited 2023 Nov 9. 17785532 10.1101/gr.6665407 PMC1987352

[14] Euesden J, Lewis CM, O’Reilly PF. PRSice: Polygenic Risk Score software. Bioinformatics. 2015;31:1466–8 Available from: https://pubmed.ncbi.nlm.nih.gov/25550326/. Cited 2023 Nov 9. 25550326 10.1093/bioinformatics/btu848 PMC4410663

[15] Ge T, Chen CY, Ni Y, Feng YCA, Smoller JW. Polygenic prediction via Bayesian regression and continuous shrinkage priors. Nat Commun. 2019; 10. Available from: https://pubmed.ncbi.nlm.nih.gov/30992449/. 10.1038/s41467-019-09718-5 PMC6467998 30992449

[16] Zhang Q, Privé F, Vilhjálmsson B, Speed D. Improved genetic prediction of complex traits from individual-level data or summary statistics. Nat Commun. 2021;12:1–9 Available from: https://www.nature.com/articles/s41467-021-24485-y. Cited 2023 Nov 9. 34234142 10.1038/s41467-021-24485-y PMC8263809

[17] Gosselin D, Skola D, Coufal NG, Holtman IR, Schlachetzki JCM, Sajti E, et al. An environment-dependent transcriptional network specifies human microglia identity. Science. 2017;356(6344). Available from: https://pubmed.ncbi.nlm.nih.gov/28546318/. 10.1126/science.aal3222 PMC5858585 28546318

[18] Tansey KE, Cameron D, Hill MJ. Genetic risk for Alzheimer’s disease is concentrated in specific macrophage and microglial transcriptional networks. Genome Med. 2018;10:1–10 Available from: https://genomemedicine.biomedcentral.com/articles/10.1186/s13073-018-0523-8. Cited 2023 Nov 10. 29482603 10.1186/s13073-018-0523-8 PMC5828245

[19] Baker E, Leonenko G, Schmidt KM, Hill M, Myers AJ, Shoai M, et al. What does heritability of Alzheimer’s disease represent? PLoS ONE. 2023;18:e0281440. 37115753 10.1371/journal.pone.0281440 PMC10146480

[20] Lambert JC, Ibrahim-Verbaas CA, Harold D, Naj AC, Sims R, Bellenguez C, et al. Meta-analysis of 74,046 individuals identifies 11 new susceptibility loci for Alzheimer’s disease. Nat Genet. 2013;45:1452–8. 24162737 10.1038/ng.2802 PMC3896259

[21] Petersen RC, Aisen PS, Beckett LA, Donohue MC, Gamst AC, Harvey DJ, et al. Alzheimer’s Disease Neuroimaging Initiative (ADNI): clinical characterization. Neurology. 2010;74:201–9. 20042704 10.1212/WNL.0b013e3181cb3e25 PMC2809036

[22] Marees AT, de Kluiver H, Stringer S, Vorspan F, Curis E, Marie-Claire C, et al. A tutorial on conducting genome-wide association studies: Quality control and statistical analysis. Int J Methods Psychiatr Res. 2018;27(2). Available from: https://pubmed.ncbi.nlm.nih.gov/29484742/. 10.1002/mpr.1608 PMC6001694 29484742

[23] Chang CC, Chow CC, Tellier LCCAM, Vattikuti S, Purcell SM, Lee JJ. Second-generation PLINK: rising to the challenge of larger and richer datasets. Gigascience. 2015;4:7. Available from: http://gigascience.biomedcentral.com/articles/10.1186/s13742-015-0047-8. 10.1186/s13742-015-0047-8 PMC4342193 25722852

[24] The 1000 Genomes Project Consortium, Auton A, Abecasis GR, Altshuler DM, Durbin RM, Bentley DR et al. A global reference for human genetic variation. Nature. 2015:68–74. Available from: http://www.nature.com/nature/journal/v526/n7571/full/nature15393. 10.1038/nature15393 PMC4750478 26432245

[25] Palmqvist S, Tideman P, Cullen N, Zetterberg H, Blennow K, Dage JL, et al. Prediction of future Alzheimer’s disease dementia using plasma phospho-tau combined with other accessible measures. Nat Med. 2021;27:1034–42 Available from: https://pubmed.ncbi.nlm.nih.gov/34031605/. Cited 2024 Apr 19. 34031605 10.1038/s41591-021-01348-z

[26] Palmqvist S, Rossi M, Hall S, Quadalti C, Mattsson-Carlgren N, Dellavalle S, et al. Cognitive effects of Lewy body pathology in clinically unimpaired individuals. Nat Med. 2023;29:1971–8 Available from: https://pubmed.ncbi.nlm.nih.gov/37464059/. Cited 2024 Apr 19. 37464059 10.1038/s41591-023-02450-0 PMC10427420

[27] Manichaikul A, Mychaleckyj JC, Rich SS, Daly K, Sale M, Chen WM. Robust relationship inference in genome-wide association studies. Bioinformatics. 2010;26:2867–73 Available from: https://pubmed.ncbi.nlm.nih.gov/20926424/. Cited 2024 Jan 4. 20926424 10.1093/bioinformatics/btq559 PMC3025716

[28] Speed D, Holmes J, Balding DJ. Evaluating and improving heritability models using summary statistics. Nat Genet. 2020;52:458–62 Available from: https://www.nature.com/articles/s41588-020-0600-y. Cited 2023 Nov 9. 32203469 10.1038/s41588-020-0600-y

[29] Lloyd-Jones LR, Zeng J, Sidorenko J, Yengo L, Moser G, Kemper KE, et al. Improved polygenic prediction by Bayesian multiple regression on summary statistics. Nat Commun. 2019;10(1):5086. Available from: https://pubmed.ncbi.nlm.nih.gov/31704910/. 31704910 10.1038/s41467-019-12653-0 PMC6841727

[30] Speed D, Cai N, Johnson MR, Nejentsev S, Balding DJ. Reevaluation of SNP heritability in complex human traits. Nat Genet. 2017;7:986–92. Available from: https://pubmed.ncbi.nlm.nih.gov/28530675/. 10.1038/ng.3865 PMC5493198 28530675

[31] Martin AR, Gignoux CR, Walters RK, Wojcik GL, Neale BM, Gravel S, et al. Human demographic history impacts genetic risk prediction across diverse populations. Am J Hum Genet. 2017;100:635–49 Available from: https://pubmed.ncbi.nlm.nih.gov/28366442/. Cited 2024 Jan 22. 28366442 10.1016/j.ajhg.2017.03.004 PMC5384097

[32] Scutari M, Mackay I, Balding D. Using genetic distance to infer the accuracy of genomic prediction. PLoS Genet. 2016;12. Available from: https://pubmed.ncbi.nlm.nih.gov/27589268/. Cited 2024 Jan 22. 10.1371/journal.pgen.1006288 PMC5010218 27589268

[33] Ruan Y, Lin YF, Feng YCA, Chen CY, Lam M, Guo Z, et al. Improving polygenic prediction in ancestrally diverse populations. Nat Genet. 2022;54:573–80 Available from: https://pubmed.ncbi.nlm.nih.gov/35513724/. Cited 2024 Jan 23. 35513724 10.1038/s41588-022-01054-7 PMC9117455

[34] Escott-Price V, Hardy J. Genome-wide association studies for Alzheimer’s disease: bigger is not always better. Brain Commun. 2022;4. 10.1093/braincomms/fcac125. Cited 2023 Dec 12. 10.1093/braincomms/fcac125 PMC9155614 35663382

[35] Fahed AC, Philippakis AA, Khera AV. The potential of polygenic scores to improve cost and efficiency of clinical trials. Nat Commun. 2022;13:1–4. Available from: https://www.nature.com/articles/s41467-022-30675-z. Cited 2023 Nov 15. 35614072 10.1038/s41467-022-30675-z PMC9132885

[36] Clark K, Leung YY, Lee WP, Voight B, Wang LS. Polygenic risk scores in Alzheimer’s disease genetics: methodology, applications, inclusion, and diversity. J Alzheimer’s Dis. 2022;89:1. Available from: https://pubmed.ncbi.nlm.nih.gov/35848019/. Cited 2023 Nov 21. 35848019 10.3233/JAD-220025 PMC9484091

[37] Choi SW, Mak TSH, Hoggart CJ, O’reilly PF. EraSOR: a software tool to eliminate inflation caused by sample overlap in polygenic score analyses. Gigascience. 2022;12:1–11 Available from: 10.1093/gigascience/giad043. Cited 2023 Dec 6. 10.1093/gigascience/giad043 PMC10273836 37326441

[38] Choi SW, Mak TSH, O’Reilly PF. Tutorial: a guide to performing polygenic risk score analyses. Nat Protoc. 2020;15:2759–72 Available from: https://www.nature.com/articles/s41596-020-0353-1. Cited 2024 Apr 17. 32709988 10.1038/s41596-020-0353-1 PMC7612115

[39] Ganna Leonenko, Maryam Shoai, Eftychia Bellou, Rebecca Sims, Julie Williams, John Hardy VE-P, Leonenko G, Shoai M, Bellou E, Sims R, Williams J, et al. Genetic risk for alzheimer disease is distinct from genetic risk for amyloid deposition. Ann Neurol. 2019;86(3):427-35. Available from: https://pubmed.ncbi.nlm.nih.gov/31199530. 10.1002/ana.25530 PMC6771864 31199530

[40] Chen Y, Hong T, Chen F, Sun Y, Wang Y, Cui L. Interplay between Microglia and Alzheimer’s Disease—Focus on the most relevant risks: APOE genotype, sex and age. Front Aging Neurosci. 2021;13:631827. 33897406 10.3389/fnagi.2021.631827 PMC8060487

[41] Wu Y, Sun Z, Zheng Q, Miao J, Dorn S, Mukherjee S, et al. Pervasive biases in proxy genome-wide association studies based on parental history of Alzheimer’s disease. Nat Genet. 2024;56:2696–703 Available from: https://www.nature.com/articles/s41588-024-01963-9. Cited 2024 Dec 11. 39496879 10.1038/s41588-024-01963-9 PMC11929606

[42] Ding Y, Hou K, Burch KS, Lapinska S, Privé F, Vilhjálmsson B, et al. Large uncertainty in individual polygenic risk score estimation impacts PRS-based risk stratification. Nat Genet. 2022;54:30–9 Available from: https://pubmed.ncbi.nlm.nih.gov/34931067/. Cited 2024 Apr 17. 34931067 10.1038/s41588-021-00961-5 PMC8758557

[43] Lo MT, Kauppi K, Fan CC, Sanyal N, Reas ET, Sundar VS, et al. Identification of genetic heterogeneity of Alzheimer’s disease across age. Neurobiol Aging. 2019;84:243.e1-243.e9 Available from: https://pubmed.ncbi.nlm.nih.gov/30979435/. Cited 2024 Jul 18. 30979435 10.1016/j.neurobiolaging.2019.02.022 PMC6783343

